# Polymer-Assisted 3D Printing of Inductor Cores

**DOI:** 10.1021/acsami.3c18956

**Published:** 2024-02-13

**Authors:** Zhidong Luo, Qi Yue, Xueyuan Li, Yuchen Zhu, Xuzhao Liu, Lee A. Fielding

**Affiliations:** † Department of Materials, School of Natural Sciences, The University of Manchester, Oxford Road, Manchester M13 9PL, U.K.; ‡ Henry Royce Institute, The University of Manchester, Oxford Road, Manchester M13 9PL, U.K.

**Keywords:** 3D printing, iron oxide nanoparticles, RAFT polymerization, inductors, ceramic inks

## Abstract

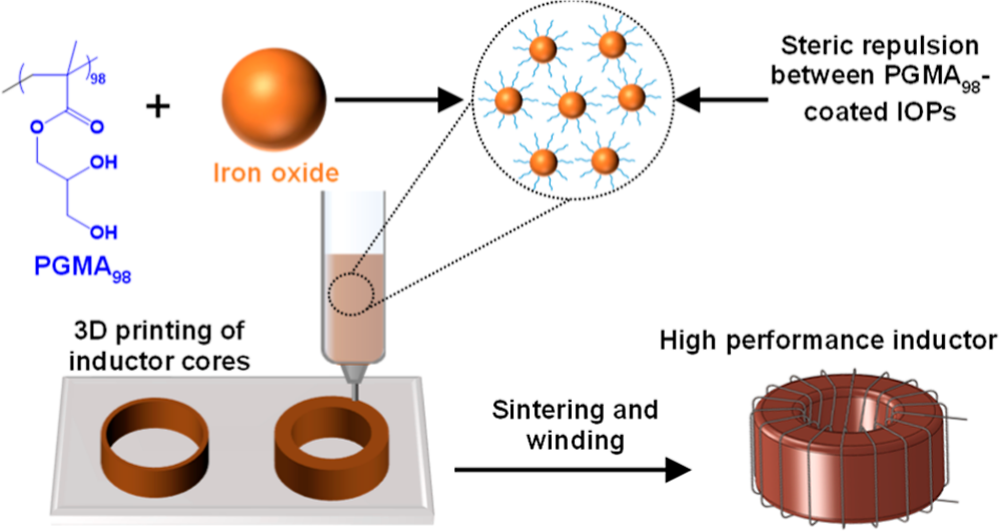

Poly(glycerol monomethacrylate) (PGMA) prepared by reversible addition–fragmentation chain transfer polymerization was investigated as an additive for high-loading iron oxide nanoparticle (IOP) 3D printable inks. The effect of adjusting the molar mass and loading of PGMA on the rheology of IOP suspensions was investigated, and an optimized ink formulation containing 70% w/w IOPs and 0.25% w/w PGMA_98_ at pH 10 was developed. This ink was successfully 3D printed onto various substrates and into several structures, including rectangles, high aspect ratio cylinders, letters, spiral- and comb-shaped structures, and thin- and thick-walled toroids. The effect of sintering on the mechanical properties of printed artifacts was investigated via four-point flexural and compressive testing, with higher sintering temperatures resulting in improved mechanical properties due to changes in the particle size and microstructure. The printed toroids were fabricated into inductors, and their electrical performance was assessed via impedance spectroscopy at a working frequency range of 0.001–13 MHz. There was a clear trade-off between electrical properties and sintering temperature due to a phase change between γ-Fe_2_O_3_ and α-Fe_2_O_3_ upon heating. Nevertheless, the optimized devices had a *Q* factor of ∼40 at 10 MHz, representing a superior performance compared to that of other inductors with iron oxide cores previously reported. Thus, this report represents a significant step toward the development of low-cost, fully aqueous, high loading, and 3D printable ceramic inks for high-performance inductors and functional devices.

## Introduction

Iron oxide nanoparticles (IOPs) are soft magnetic particles with high surface areas and are widely used in water treatment,^1^ diagnostic imaging,^2^ drug delivery,^3^ and inductive cores.^4^ An inductor is an electronic component that stores energy within a magnetic field as an electric current runs through it. It also counteracts changes in the current by generating a voltage that opposes the direction of the electric current. Ongoing challenges in the development of inductors include size minimization and increased shape complexity.^4,5^ Ferrite ceramics, noted for their high resistivity and superior magnetic properties, serve as the essential materials in inductor core manufacturing.^6^ However, it is difficult to fabricate these ferrite ceramics with thin walls, porous structures, or complex shapes by traditional technologies owing to their hardness and brittleness.^7,8^ In contrast, additive manufacturing (AM) fabricates objects layer-by-layer according to three-dimensional (3D) model data and allows the production of complex shapes precisely and rapidly.^8^ Thus, AM of inductor cores using IOPs is a promising route to address these manufacturing issues.

Extrusion-based direct ink writing (DIW), a sub-branch of AM, extrudes concentrated suspension inks through a printing nozzle to form desired shapes.^4^ Suitable rheological properties are an essential requirement of ceramic inks.^9^ These inks should have shear thinning behavior to make them flow during extrusion and a large enough storage modulus (*G*′) to retain their shape under gravity after extrusion.^4,7,10^ However, there are always trade-offs between the storage modulus and viscosity when DIW inks are formulated.^11^ High storage modulus inks are thick and have high viscosities under shear thinning conditions. This means that they typically have poor flowability, and printed objects have rough and textured surfaces.^12^ Conversely, low storage modulus inks have low viscosity and good flowability, but their shape retention ability is often poor. Organic additives such as dispersants,^13^ viscosifiers,^14^ binders,^15^ stiffeners,^16^ surfactants,^13^ and diluents^11^ are commonly utilized during formulation to adjust the rheological properties of DIW inks. Currently, additive loadings are usually relatively high (5–30% w/w), potentially resulting in undesired shrinkage and internal defects of final parts if the additives need to be removed by postprocessing.^8^ Consequently, there is a pressing need for polymer additives that can efficiently modify the rheology of inks and support high particle loadings at very low doses.

One such approach to achieve this is the use of copolymers. For example, Wang et al., demonstrated that a poly(acrylic acid-*b*-*N*-isopropylacrylamide) (PAA–PNIPAM) block copolymer dispersant allowed the formulation of high loading (40 v/v %) aluminum oxide (Al_2_O_3_) inks at relatively low polymer concentrations (0.08% w/w).^100^ It was shown that pH and temperature had a strong impact on the stability and rheology of the prepared inks. However, cellulose was required as an additional plasticizer in these formulations to achieve good interlayer adherence when printing this ink. Similarly, statistical copolymers of methyl acrylate-esterified poly(ethylene glycol) (MAPEG), *N*-[3(dimethylamino)propyl]methacrylamide (DMAPMA), and acrylic acid (AA) allowed the formulation of Fe_3_O_4_ magnetic inks with remarkably high Fe_3_O_4_ loadings (81% w/w) at relatively low additive concentrations (1.15% w/w).^11^ It was shown that the ratio of MAPEG/AA/DMAPMA in the copolymer impacted ink viscosity to a large extent due to the varying degrees of electrostatic and steric repulsion imparted to the Fe_3_O_4_ particles upon their absorption. In both cases, the copolymer dispersant played a pivotal role in tailoring the ink stability and rheology, allowing high loading ink preparation.

IOPs typically have surfaces containing hydroxyl groups that can act as anchoring points for polymers containing carboxyl,^17^ amine,^11^ hydroxyl,^19^ and other functional groups. Reported (co)polymers that contain these functional groups,^11,18,20,21^ therefore have the potential to be used as additives in high IOP loading inks to improve their stability and rheology.^22−24^ However, the additives commonly reported for this purpose are relatively complex synthetically, can be ill-defined, and their behavior can vary as a function of pH and other printing conditions. Furthermore, other additives in addition to the polymer dispersant are often needed, complicating ink formulation.^25^ It is therefore desirable for nonionic, relatively simple polymer additives with a controlled structure and molecular weight to increase the stability and improve the rheology of inks to be developed and studied.

**Figure 1:**
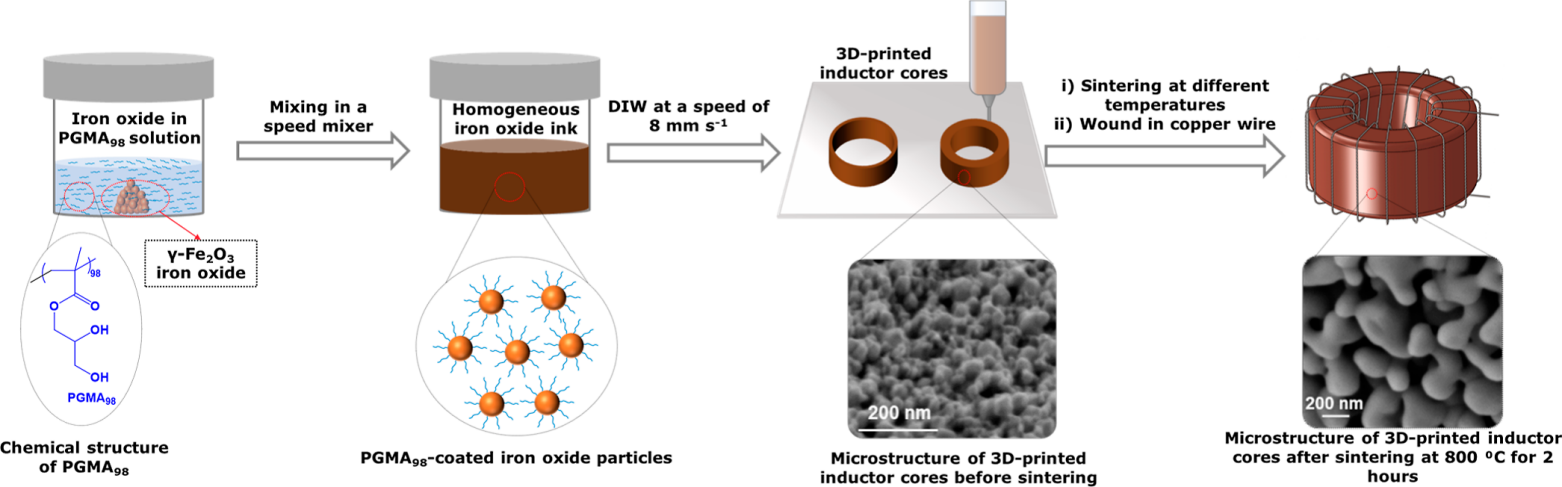
Preparation of IOP inks using PGMA_98_ as an additive and subsequent fabrication of inductor cores via DIW.

Herein, a series of poly(glycerol monomethacrylate (GMA)) (PGMA) homopolymers were prepared via reversible addition–fragmentation chain transfer (RAFT) polymerization and investigated as nonionic, water-soluble additives for the formulation of aqueous IOP-based DIW inks (Figure 1). This method is more controllable and simpler in terms of synthetic ease, chemistry used, and potential scalability than previously reported approaches for relatively complex copolymers. γ-Fe_2_O_3_ was selected as the functional material for these inks as, compared to other kinds of iron oxide, γ-Fe_2_O_3_ has lower coercivity, higher electrical resistivity, and good thermal stability, making it a good candidate to form inductor cores for high frequency applications. The rheology of high concentration γ-Fe_2_O_3_ IOP dispersions at relatively low polymer loadings was studied systematically, with the effect of pH, PGMA molecular weight, and dosage of the polymer being investigated. The printability of an optimized PGMA-containing IOP ink was demonstrated, and the properties of the printed structures were investigated after various post processing steps. Four-point flexural and compressive testing were applied to measure the mechanical properties of air-dried and sintered structures, and X-ray diffraction (XRD) was used to demonstrate the iron oxide phase changes caused by sintering. Finally, thin-walled and thick-walled toroidal magnetic cores were printed, and their electrical performance characterized by impedance spectroscopy (IS). The relative permeability and quality factor (*Q* factor) of the inductors prepared by this route were subsequently compared to related examples from the literature.

## Experimental Section

### Materials

All reagents, unless otherwise noted, were procured from Merck (UK) and used as received. GMA was generously donated by GEO Specialty Chemicals (UK). 4-Cyano-4-(2-phenylethane sulfanylthiocarbonyl)sulfanylpentanoic acid (PETTC) was prepared in-house using previously published methods.^19^ Iron(III) oxide nanoparticle powder (20–40 nm average particle size) was purchased from Alfa Aesar (UK) and used as received. Ethanol (95%) was obtained from Fisher Scientific (UK) and used as received. Deionized water with a resistivity of 18.2 MΩ cm was used in all experiments.

### Synthesis of the PGMA Additive

PGMA was synthesized via RAFT solution polymerization following an established method (Figure S1).^26−30^ A typical protocol targeting a degree of polymerization (DP) of 100 is as follows. GMA (10.0 g, 62 mmol), PETTC (0.21 g, 0.62 mmol), and azobis(4-cyanovaleric acid) (ACVA, 0.0347 g, 0.124 mmol) were dissolved in anhydrous ethanol (10.2 g, previously purged with nitrogen for 20 min) within a 100 mL round-bottomed flask. This flask was subsequently sealed and purged with N_2_ for 30 min. Then, the flask was immersed in a preheated oil bath at 70 °C for 2 h. After 2 h, the flask was taken out from the oil bath and immersed in an ice bath to stop polymerization. The obtained polymer solution was purified using dialysis against water (MWCO = 1000 g mol^–1^) and then freeze-dried. The final DP was determined by ^1^H NMR using deuterium oxide (D_2_O) as a solvent (Figure S2), and the molar mass distribution was measured using gel permeation chromatography (GPC) (Figure S3).

### Preparation of Iron Oxide Inks

To illustrate the preparation of an ink with 70% w/w IOP loading and 0.25% w/w PGMA_98_ loading at pH = 10, 20 g of IOPs were transferred into a 60 mL jar. 0.05 g of PGMA_98_ was dissolved in 8.57 g of deionized water, and the pH was adjusted to 10 by adding 0.1 and 0.01 M KOH solution. This solution was then injected into the jar containing IOPs. This jar was mixed using a speed mixer (Synergy Devices Ltd., Bucks, UK) at 400 rpm for 1 min, 1000 rpm for 1 min, 1200 rpm for 2 min, 1800 rpm for 2 min, 2000 rpm for 1 min, and 400 rpm for 1 min to form a homogeneous IOP ink. Other reported inks were prepared through the same procedure by changing the pH and loading of the IOP and polymer.

### Direct Ink Writing

Various structures were printed onto aluminum, cardboard, blue tissue paper and nitrile lab glove substrates by a robot printer (I&J7300R-LF Robots, I&J Fisnar Inc. Wayne, NJ, USA, Figure S8). The diameter of the print head, and thus the line width resolution, was 0.84 mm, the nozzle head speed was fixed at 8 mm s^–1^, and the layer thickness was set to 0.8 mm. After printing, the green bodies were dried in air for at least 12 h before being removed from the substrate.

### Sintering

The dried samples were sintered at different temperatures (400, 600, and 800 °C) in air using a furnace (Nabertherm Muffle Furnace LT 1300 Series with B410 Controller). The heating profiles are shown in Figure 4a.

### Rheology of Iron Oxide Inks

Rheological measurements were performed using a HAAKE MARS iQ Rheometer equipped with a flat titanium plate of 35 mm diameter. In dynamic testing, the oscillation frequency was set to 1.592 Hz, and the strain was changed from 0.0035 to 3 to determine the storage and loss moduli of the inks. In steady-state viscosity measurements, the shear rate was changed from 0.100 to 100 s^–1^.

### Mechanical Testing

An Instron 3344L3928 2 kN universal testing system with different functional fixtures was used for four-point flexural and compressive tests. For four-point flexural testing, a four-point bend fixture (Figure S9a) was assembled with the testing system. Printed rectangle blocks (30 × 15 × 2.8 mm) with different postprocessing were used as samples. For compressive testing, a compressive test fixture (Figure S9b) was used, and printed thin-walled toroidal cores (8 × 20 × 3 mm, height × outer diameter × thickness) with different postprocessing were used as samples. The flexural modulus and compressive modulus were determined by fitting the stress–strain curves.

### Field Emission Scanning Electron Microscopy

Scanning electron microscopy (SEM) images were captured using a Zeiss Merlin FEG-SEM or a TESCAN Mira3 FEG-SEM. All samples were coated with a 5 nm thickness Au/Pd layer and imaged using a relatively low accelerating voltage (2–5 kV) and beam current (∼67 pA) to reduce surface charging effects. The particle size distribution was determined by counting the longest dimension of 50 randomly selected particles.

### Density Measurements

Density measurements were conducted using an A&D HR-150AZ analytical balance equipped with an A&D density determination kit to determine the density of green bodies and sintered bodies according to Archimedes’ method. Ethanol was used as the medium. Before testing, the sample surfaces were sprayed with a very thin layer of an acrylic-based resin and dried in air to avoid liquid penetration.

### Impedance Spectroscopy

The impedance, *Q* factor, and inductance were measured by using a 41921A LF impedance analyzer. All inductor cores were wound in a 28 American Wire Gauge (AWG) copper wire to form 20-turn coils and connected to the impedance analyzer. During measurements, an applied voltage was set to 1 V and the frequency was swept from 1 kHz to 13 MHz.

## Results and Discussion

### Rheology of PGMA-Containing IOP Inks

PGMA is a nonionic, water-soluble, polymer that was hypothesized to be able to absorb onto the surface of IOPs through hydrogen bonding or interactions between the or 1,2-diol on the polymer and Fe atoms to form a five-membered chelate ring.^18^ Initially, a PGMA homopolymer with a DP of 98 was prepared by RAFT solution polymerization in ethanol (Figures S1 and S2). This polymer had an *M*_n_ of 18,000 g mol^–1^ and an *M*_w_/*M*_n_ of 1.19, as determined by GPC analysis (Figure S3).

**Figure 2:**
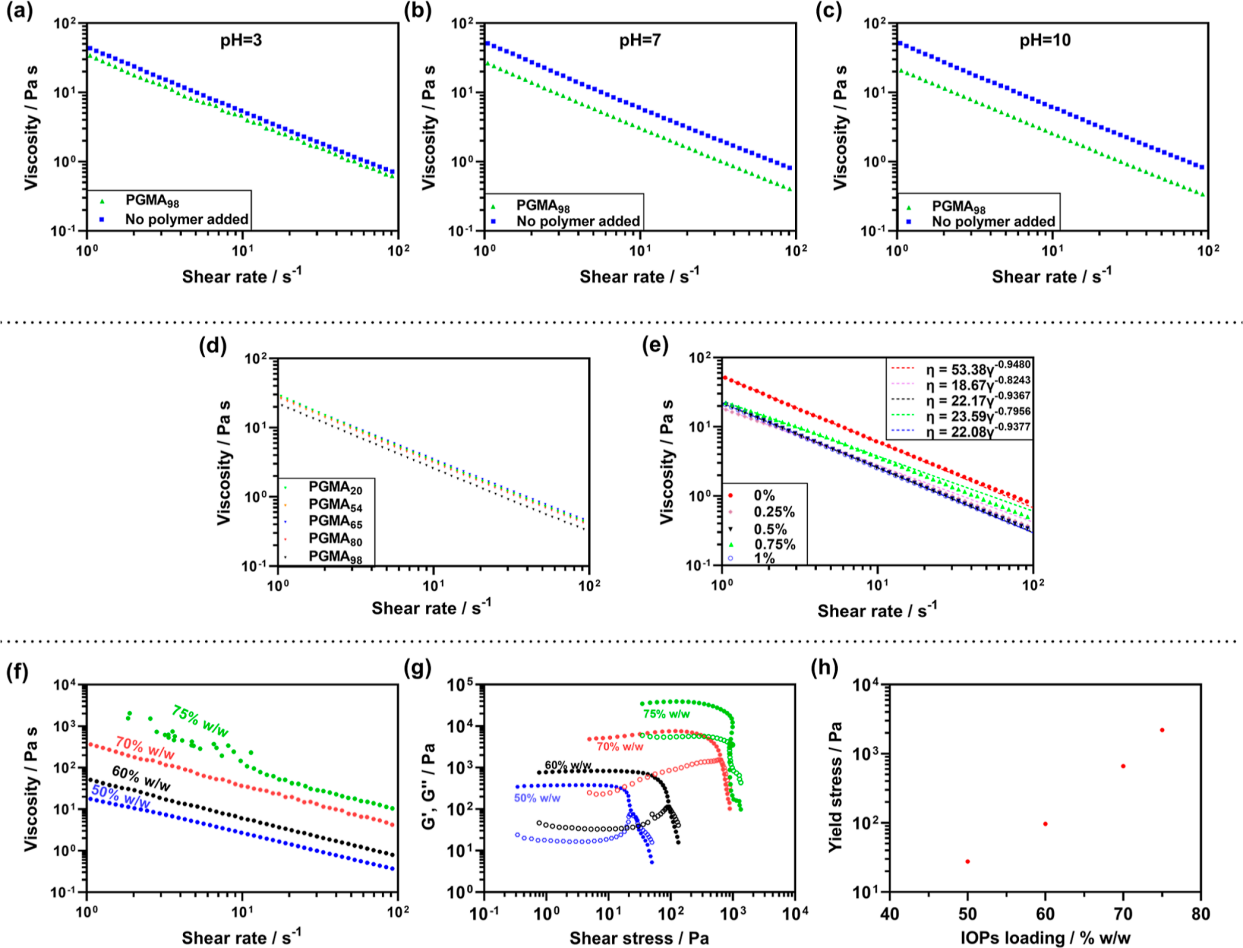
(a–c) Viscosity vs shear rate of 50% w/w IOP suspensions with (green triangles) and without (blue squares) 0.5% w/w PGMA_98_, based on IOP concentration, added at (a) pH = 3, (b) pH = 7, and (c) pH = 10. (d) Change in the viscosity of 50% w/w IOP suspensions in the presence of PGMA_*x*_ polymers with differing degrees of polymerization. (e) Change in the viscosity of 50% w/w IOP suspensions in the presence of 0.25–1.0% w/w PGMA_98_, based on IOP concentration. (f–h) Rheology of IOP inks at pH 10 with 0.25% w/w PGMA_98_, based on IOP concentration, at different IOP loadings (50, 60, 70, and 75% w/w): (f) viscosity as a function of shear rate; (g) storage modulus (*G*′) and loss modulus (*G*″) as a function of shear stress (solid dots are *G*′ and hollow dots are *G*″); and (h) determined yield stresses, i.e., the point where *G*′ = *G*′′.

Initially, this polymer was mixed with 50% w/w IOP dispersions at 0.5% w/w PGMA_98_, based on IOPs, using a high-speed mixer. The pH of these dispersions was varied (pH 3, 7, and 10), and the viscosity was compared among IOP dispersions without added PGMA_98_ (Figure 2a–c). In all cases, the IOP dispersions had shear thinning behavior. However, the addition of PGMA_98_ significantly decreased the viscosity of the dispersions to approximately 1/3 of the viscosity without PGMA_98_. As a nonionic polymer, PGMA_98_ absorbs onto the IOP surfaces and introduces steric repulsion between the IOPs. This steric hindrance lubricates the flow of the IOPs, consequently leading to a marked reduction in viscosity. The amount of the polymer absorbed onto the IOPs for PGMA_98_ loadings between 0.75 and 5.8% w/w was investigated by thermogravimetric analysis (TGA) after two washing cycles (Figure S5). The mass loss that occurred between 200 and 350 °C was attributed to pyrolysis of PGMA_98_, and as expected, increasing the amount of PGMA_98_ added resulted in more polymer being absorbed. However, in all cases, the absorption ratio was less than 1.0, meaning that not all of the polymer remained absorbed to the IOPs after being challenged with a washing cycle. Nevertheless, the zeta potential and hydrodynamic diameter of 0.1% w/w IOP dispersions were not significantly affected by the addition of PGMA_98_ (Figure S4). For both pristine and PGMA_98_-containing IOP dispersions, the zeta potential was found to be positive at low pH and transitioned to being negative above approximately pH 7.5. Near the isoelectric point, evidence of particle aggregation was observed by dynamic light scattering (DLS) for both pristine and PGMA_98_-containing dispersions. While the nonionic nature of PGMA was not expected to affect the zeta potential of the IOPs, the observed aggregation at ∼pH 7.5 was somewhat unexpected as it was believed that the PGMA_98_ would impart steric stabilization to the IOPs, preventing aggregation. Nevertheless, despite this observation, the rheological behavior at high IOP concentrations remained consistent and showed pH-independent behavior, and the pH of the dispersion did not dramatically affect the measured viscosity, highlighting the versatile nature of this polymeric dispersant. Furthermore, the lowest measured viscosity was for dispersions at pH 10, and thus, all formulations discussed herein were kept constant at this pH.

A series of PGMA homopolymers with differing *M*_n_ values were prepared (Table S1) to assess the effect of molecular weight on ink rheology. The viscosities of dispersions with 0.5% w/w PGMA_*x*_, based on IOP loading (where *x* = 20 to 98), are shown in Figure 2d. The molecular weight of PGMA_*x*_ did not significantly affect the measured viscosity, and all dispersions retained their shear thinning behavior. The dispersion with the lowest viscosity was obtained when PGMA_98_ was added. This is likely due to more effective steric repulsion being imparted by the polymer with the highest molecular weight studied in this investigation.

The effect of the PGMA_98_ dosage (0–1.0% w/w, based on IOP concentration) on the viscosity of 50% w/w IOP dispersions was investigated (Figure 2e). These dispersions displayed a significant decrease in viscosity compared to pristine IOPs. Interestingly, varying the dosage between 0.25 and 1.0% w/w PGMA_98_ had relatively little effect on the viscosity of the dispersion. The viscosity–shear rate dependence was fitted using a power law, μ = *K*γ̇^*n*–1^,^31^ where *K* is the flow consistency index, γ̇ is the shear rate, and *n* is the flow behavior index. Dispersions with the lowest polymer loading tested (0.25% w/w PGMA_98_) had the lowest flow consistency index (18.67) and the second lowest flow behavior index (0.1757, Figure 2e inset), indicating that this dispersion possessed the lowest resting viscosity and the second lowest shear thinning behavior. Thus, this PGMA_98_ loading was fixed for subsequent ink formulation tests.

Dispersions with increasing IOP loadings (50–75% w/w) were prepared and analyzed via rheology to investigate the maximum concentration of IOPs that could be used in a DIW ink. As shown in Figure 2f, all samples were shear thinning, but the viscosity of the dispersions increased by nearly 2 orders of magnitude as the IOP loading was increased from 50 to 75% w/w. Additionally, all samples demonstrated elastic behavior (*G*′ > *G*″) up to the yield point (*G*′ = *G*″, Figure 2g).^32^

For successful DIW, the yield stress of the ink should be lower than the maximum shear stress at the wall of the printhead to ensure controllable flow of the ink. The maximum shear stress is determined using τ = (Δ*P*/2*L*)*r*, where τ is the maximum shear stress, Δ*P* is the pressure applied at the nozzle, *L* is the length of the nozzle, and *r* is the radius of the nozzle.^11,33,34^ For the printer used in this work, the maximum shear stress at the wall of the nozzle was calculated to be ∼723 Pa (Δ*P* = 43,750 Pa, *r* = 0.42 mm, and *L* = 12.7 mm). However, due to the non-Newtonian nature and dynamic conditions during printing, the actual shear stress achievable using this setup would be lower than 723 Pa in practice. As shown in Figure 2h, the yield stress of the prepared IOP inks increased from ∼28 Pa to 2.2 kPa as the IOP loading was varied from 50 to 75% w/w. Since the yield stress of the 75% w/w IOP ink was higher than the maximum shear stress for the printer, this ink could not be used in further studies. However, this ink would be suitable for printers with different geometries or higher maximum pressures, representing a very high solids loading ink formulation, comparable to that of other reported IOP formulations,^4,7,8,11,35^ enabled by this PGMA_98_ dispersant.

The yield stress of the 70% w/w IOP ink was lower than the maximum shear stress for our printer and had a high storage modulus (∼7500 Pa) at low shear stress, indicating that it would have good shape retention upon printing. Thus, this formulation (70% w/w IOP with 0.25% w/w PGMA_98_, based on IOP concentration, at pH 10) was chosen for the following printing studies.

### 3D Printing and Sintering

**Figure 3:**
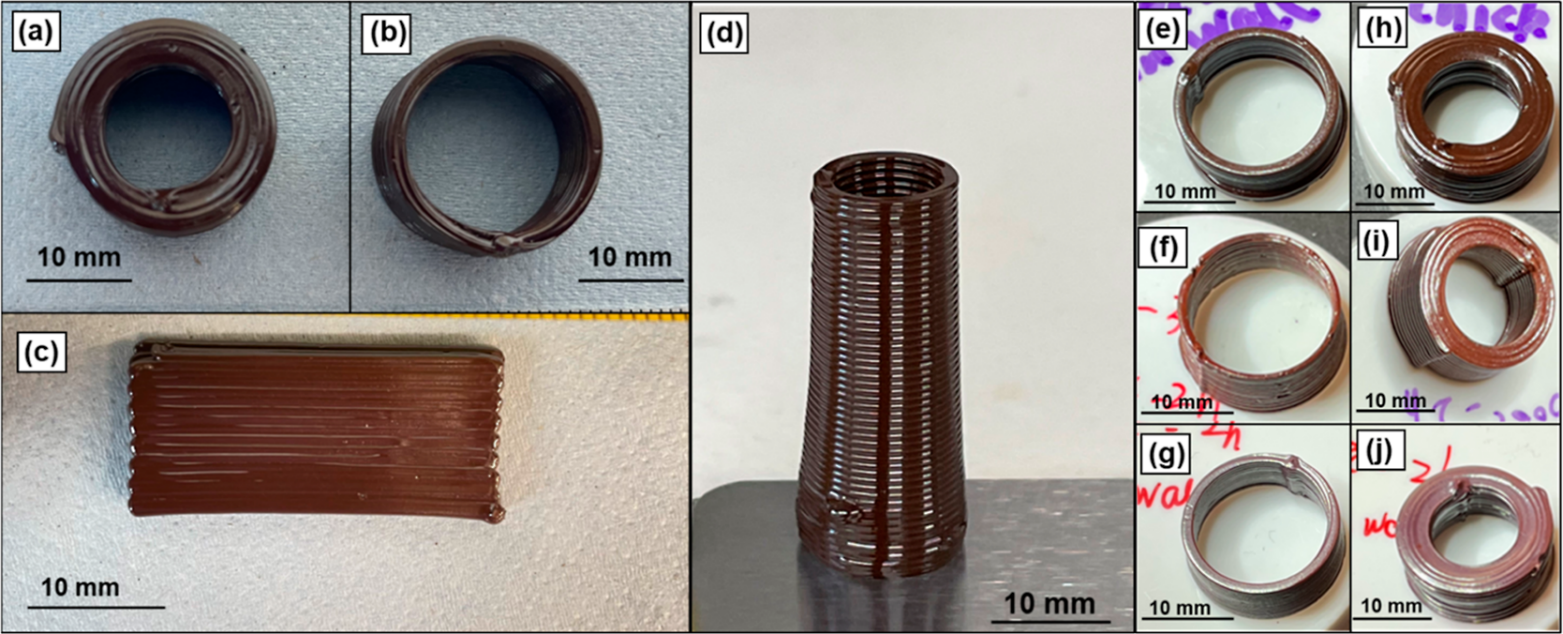
Photographs of as-printed shapes after DIW of formulated IOP inks: (a) thick-walled toroidal inductor core; (b) thin-walled toroidal inductor core; (c) rectangular block; and (d) high height-to-wall thickness ratio (32:1) cylinder. (e–j) Photographs of thin-walled (left) and thick-walled (right) toroidal inductor cores after sintering at different temperatures for 2 h: (e,h) 400 °C, (f, i) 600 °C, and (g, j) 800 °C.

Four distinct shapes were printed using the formulated IOP ink onto aluminum substrates and dried at room temperature: (i) thick-walled toroidal cores (Figure 3a, dimensions: 8 × 20 × 3 mm, height × outer diameter × wall thickness); (ii) thin-walled toroidal cores (Figure 3b, dimensions: 8 × 20 × 1 mm, height × outer diameter × wall thickness); (iii) rectangular blocks (Figure 3c, dimensions: 30 × 15 × 2.8 mm, length × width × height), and (iv) a cylinder with a high height-to-wall thickness ratio of 32:1 (Figure 3d, 50 layers, dimensions: 32 × 10 × 1 mm, height × outer diameter × wall thickness). Furthermore, to demonstrate that this ink and 3D printing method can readily fabricate more complex shapes that would be difficult to simply mold, a spiral-shaped artifact with a small gap between the printed spiral (Figure S8e) and a comb-like structure with right-angled corners (Figure S8f) were printed onto aluminum substrates. In addition, the printability onto different substrates was demonstrated by printing lettering onto cardboard, paper, and nitrile rubber (Figures S8b–d). All samples were printed successfully from CAD files and retained their shapes permanently. The printed thin- and thick-walled toroidal cores were used for subsequent impedance and compressive testing, and the printed rectangular blocks were used for four-point flexural testing. The 50-layer cylinder was printed successfully without collapse or deformation, which demonstrated the excellent shape retainability of this IOP ink formulation during and after printing. However, at higher height-to-wall thickness ratios (>51:1), deformation of the top layers was observed during printing due to the cylinder being shaken from the motion of the printing plate. In practice, inductors with this extreme geometry would not be fabricated, and as demonstrated in Figure S8, complex shapes can be readily prototyped with this ink formulation.

**Figure 4:**
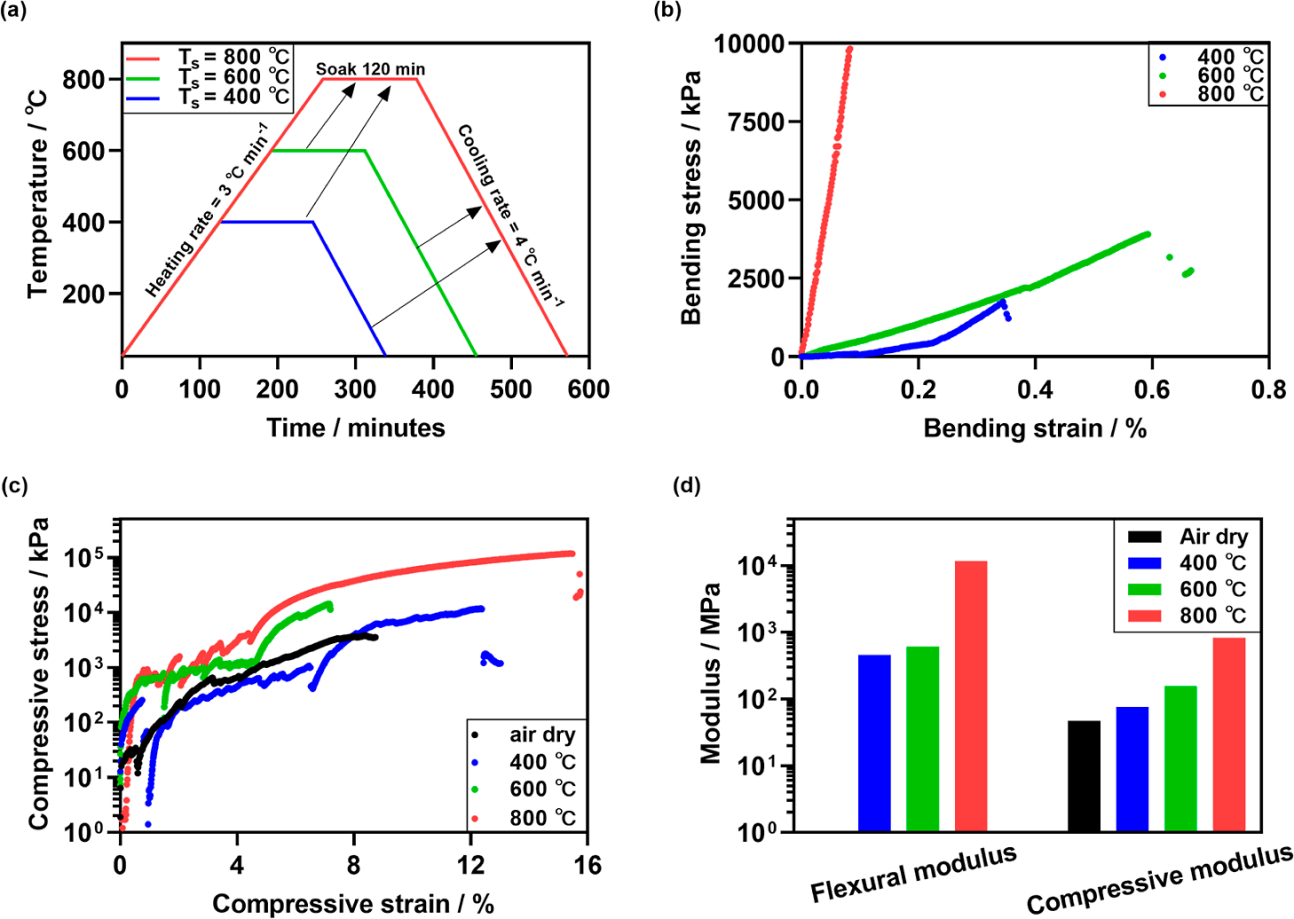
(a) Sintering profile for printed artifacts. (b) Four-point flexural test data for 3D printed rectangular blocks sintered at different temperatures. (c) Compressive testing data for 3D printed thin-walled toroidal inductor cores sintered at different temperatures. (d) Summary of flexural and compressive moduli determined for 3D printed samples sintered at different temperatures.

The as-printed structures were not able to be used as inductor cores because they still contained water. Thus, the printed structures were air-dried at room temperature for at least 12 h before further processing. However, the air-dried thin-walled toroidal cores were easily broken while being wound in copper wire, and the air-dried rectangular blocks easily disintegrated during transportation. The weak mechanical properties are likely due to shrinkage-induced defects during air-drying. Therefore, suitable postprocessing was required to improve the mechanical properties of printed samples. Sintering is a convenient, well-developed, and scalable technology commonly applied for postprocessing of 3D-printed artifacts. Thus, the 3D-printed green bodies were sintered to enhance their mechanical properties according to the heating profiles in Figure 4a.

The mechanical properties were measured using four-point bending and compressive testing equipment (Figure S9 and Table S2). In four-point bending testing (Figure 4b), the sintered rectangular blocks had maximum bending stresses of ∼1750, 3890, and 9830 kPa for samples sintered at 400, 600, and 800 °C, respectively, with the flexural moduli increasing with sintering temperature (Figure 4d). The samples sintered at 400 and 600 °C started to crack at 0.34 and 0.59% bending strain, and crack propagation continued as the bending strain increased further (e.g., up to a strain of 1.8% for the sample sintered at 600 °C). The sample sintered at 800 °C failed immediately and completely at 0.08% bending strain. Similarly, the compressive moduli (Figure 4d) and maximum compressive stress withstood for thin-walled toroids increased with increasing sintering temperature (Figure 4c). Specifically, samples failed at stresses of 3490, 11,680, 13,880, and 118,210 kPa for the air-dried sample and samples sintered at 400, 600, and 800 °C, respectively. Furthermore, while the air-dried sample had a compressive modulus of 3.5 MPa, the compressive modulus improved to 76, 157, and 832 MPa with increasing sintering temperature. Thus, sintering improved both the stiffness and the strength of the printed samples.

**Figure 5:**
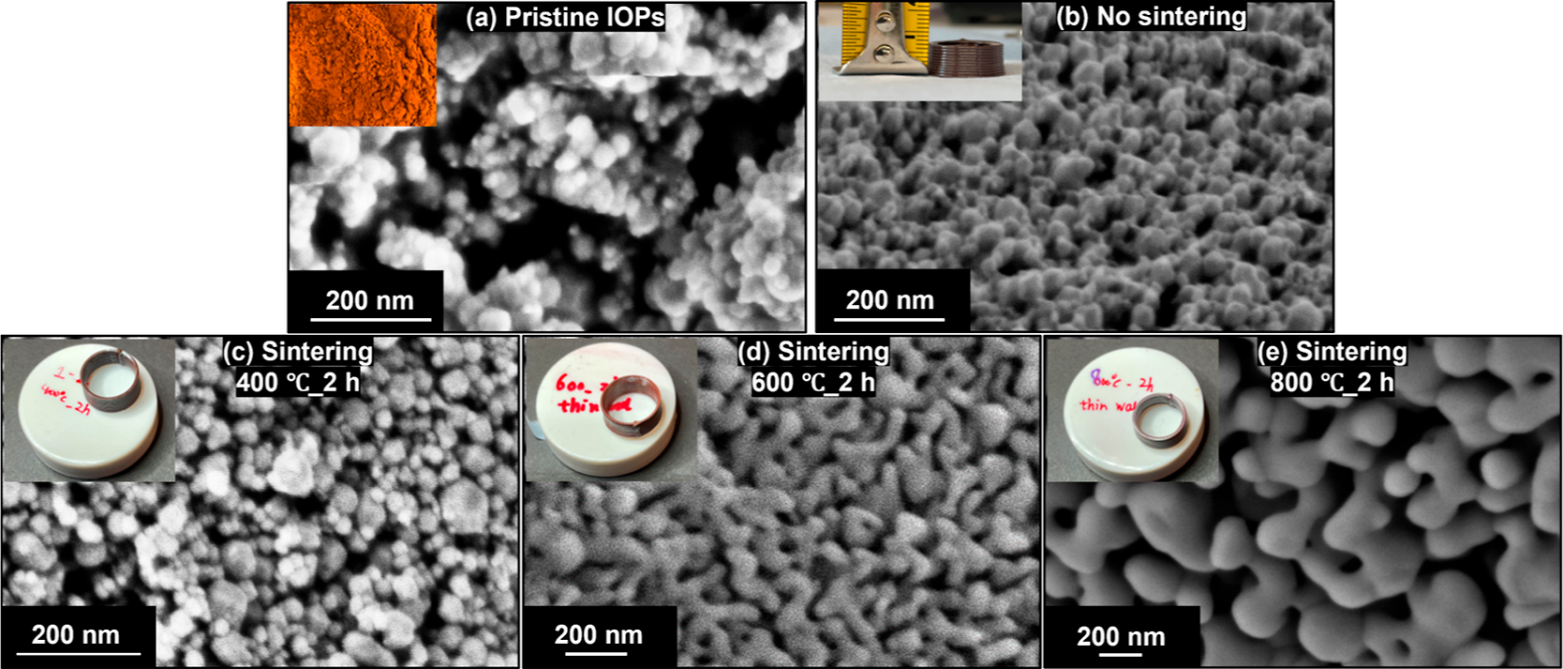
SEM images of (a) as-received IOP powder^I^ and outer surfaces of 3D printed thin-walled toroidal cores sintered under different conditions: (b) no sintering^I^; (c) 400 °C for 2 h^I^; (d) 600 °C for 2 h^II^; and (e) 800 °C for 2 h^II^. Insets show digital photographs of the objects imaged. Images were captured by I. a Zeiss Merlin FEG-SEM and II. a TESCAN Mira3 FEG-SEM.

The reason for these observed improvements can be attributed to growth and fusion of the IOPs upon sintering, as well as a reduction in the total porosity.^36−38^ Thus, SEM was utilized to observe the changes in the IOP size and sample microstructure before and after sintering (Figure 5). The particle size of the pristine IOPs was 29 ± 9 nm (Figure S6a), and as expected, this was not significantly affected by printing and air-drying (Figure S6b). Comparing Figures S6b and S6c, the particle size did not significantly increase after sintering at 400 °C (37 ± 20 nm). Furthermore, samples sintered at 400 °C for 2 h (Figure 3e,h) did not shrink, and no color change was observed. This is because 400 °C is not a high enough temperature to facilitate the necessary diffusion processes that lead to particle growth and densification, which are critical in causing shrinkage to occur during sintering. However, the IOPs did become more uniform and the large voids apparent in the air-dried sample disappeared (Figure 5c), resulting in the moderate enhancement of mechanical properties observed (Figure 4).^39^ Sintering at 600 and 800 °C increased the size of the IOPs significantly to 188 ± 71 nm at 600 °C (Figure S6d) and 458 ± 123 nm at 800 °C (Figure S6e), further enhancing the mechanical properties. These samples underwent significant shrinkage (Figure 3), with more significant shrinkage and color fading for samples sintered at 800 °C. Densification of these samples due to particle size growth^40^ was confirmed by measuring the sample densities after these different postprocessing temperatures, with determined densities of 2.39 g mL^–1^ (air-dried); 2.32 g mL^–1^ (400 °C); 2,47 g mL^–1^ (600 °C); and 3.64 g mL^–1^ (800 °C). Importantly, all sintered samples retained their shape and did not crack.

### Electrical Performance of Inductor Cores

**Figure 6:**
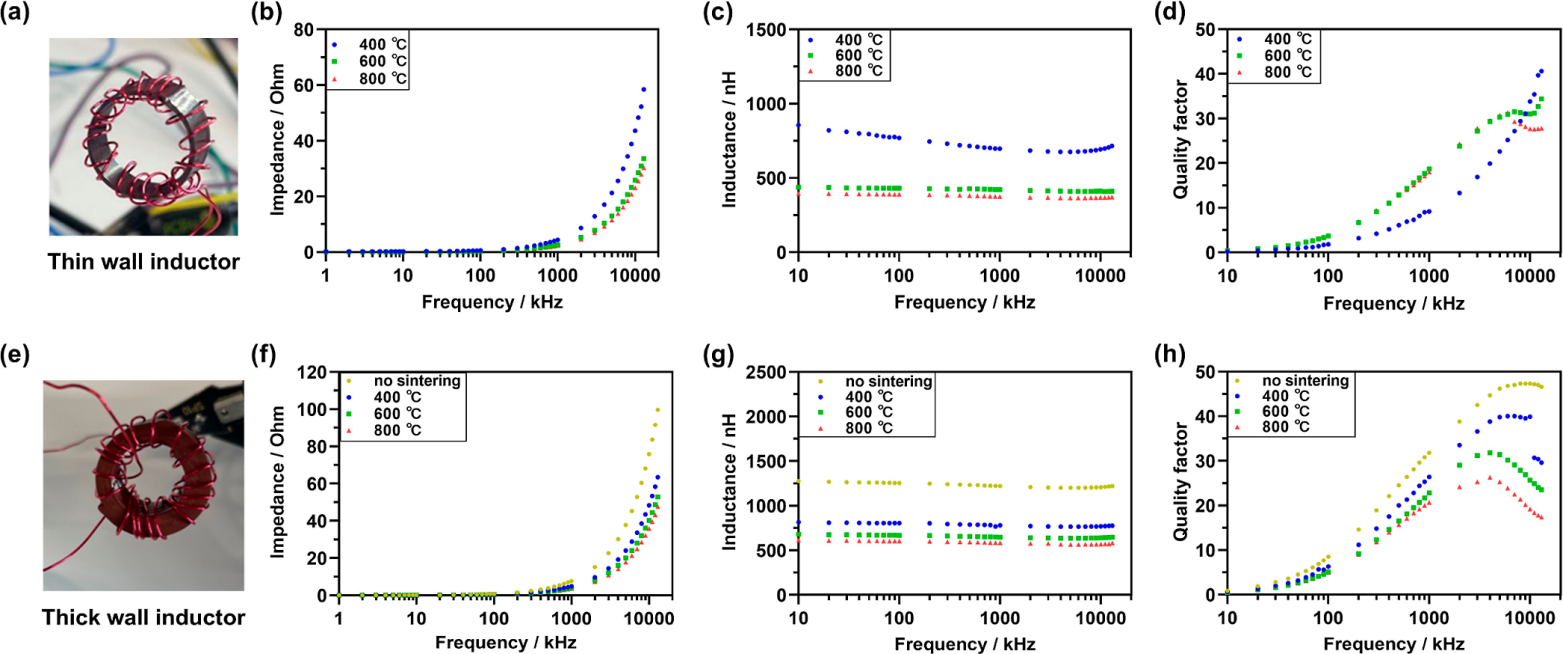
Impedance testing of inductors with 3D printed IOP toroidal cores. Digital photographs of a thin-walled (a) and a thick-walled (e) toroid wrapped in copper wire. (b,f) Impedance, (c,g) inductance, and (d,h) quality factor of inductors as a function of frequency. Data for thin- and thick-walled cores are shown on the top and bottom rows, respectively. Sintering was conducted following the profiles shown in Figure 4a.

γ-Fe_2_O_3_ is a soft magnetic material that is easily magnetized and demagnetized.^41^ This property makes γ-Fe_2_O_3_ an attractive choice for inductor core fabrication. To demonstrate the electrical properties of the printed toroidal cores described herein, each core was wound with 20 turns of 26 AWG copper wire (Figure 6a,e), connected to an impedance analyzer, and the impedance, inductance, and *Q* factor were measured as a function of working frequency (1 kHz to 13 MHz) (Figure 6, Table 1). At low frequencies (<100 kHz), the measured impedance was near zero in all cases (Figure 6b,f). As the working frequency was ramped up, the impedance of the inductors rapidly increased. Higher sintering temperatures resulted in lower impedance values, with the impedance of thin-walled inductors reducing from 59 to 30 Ω at 13 MHz as the sintering temperature was increased from 400 to 800 °C (Figure 6b and Table 1). As expected, the impedances of the thick-walled inductors were higher than that of their thin-walled counterparts but had the same trend as a function of the sintering temperature (Figure 6f and Table 1). The inductance of all of the prepared inductors was relatively frequency independent in the frequency range studied (Figure 6c,g). Higher sintering temperatures resulted in lower inductance values, dropping from 716 to 370 nH for thin-walled inductors and from 776 to 582 nH for thick-walled inductors as the sintering temperature increased from 400 to 800 °C (Table 1).

**Table 1:** Electrical Properties of 3D-Printed IOP Inductors with Toroidal Cores

sample^a^		impedance^b^/Ω	inductance^c^/nH	maximum *Q* factor^d^	resonance frequency^e^/MHz
thin-walled toroidal core inductors	400 °C	59	716	41	>13^f^
	600 °C	34	411	34	>13^f^
	800 °C	30	370	29	7
thick-walled toroidal core inductors	air-dried	100	1220	47	10
	400 °C	63	776	40	10
	600 °C	53	646	32	4
	800 °C	48	582	26	4

a Inductor cores were 3D printed using PGMA_98_-containing IOP inks. Samples were sintered at for 2 h at the temperature indicated.

b Impedance measured at 13 MHz.

c Inductance measured at 13 MHz.

d Highest recorded *Q* factor.

e Frequency where the *Q* factor reached the highest value.

f The *Q* factor was still increasing at 13 MHz.

The *Q* factor of an inductor represents its efficiency in terms of inductive reactance (energy storage) versus resistance (energy dissipation) at a given frequency.^42−44^ A higher *Q* factor value indicates that the inductor has a lower energy loss, making it more efficient. At low frequencies, the *Q* factor is generally low because the inductive reactance of the core is small compared to the resistance of the coil. As frequency increases, the *Q* factor increases due to growing inductive reactance. However, at high frequencies, the skin effect^45,46^ intensifies, increasing the resistance within the coil, and core loss^46−48^ becomes significant. These influences start to reduce the inductive reactance, leading to a resonant frequency where the inductor behaves more capacitive, and this frequency is called the resonance frequency.^42−47,49^ For the IOP-based inductors fabricated herein, the measured *Q* factors for the thick-walled inductors increased from 0 at 1 kHz to maximum values of 47 (10 MHz, air-dried), 40 (10 MHz, 400 °C), 32 (4 MHz, 600 °C), and 26 (4 MHz, 800 °C) (Figure 6h and Table 1). However, the thin-walled inductors with cores sintered at 400 and 600 °C did not reach their resonance frequencies before 13 MHz, so the expected decrease in the *Q* factor at high frequency was not observed (Figure 6d). Comparing thick- and thin-walled cores sintered at same temperature; at a constant voltage, thicker inductor cores provide a greater path for magnetic flux, resulting in higher impedance, inductance, and *Q* factor at 10 MHz (Table 1). Additionally, the thicker core size contributes to a decreased resonance frequency due to added parasitic capacitance and core loss (Table 1).^42,46,48^

The thick-walled inductor with an air-dried core had the best electrical performance (Table 1). However, this sample was not mechanically robust enough to be of practical use. Unfortunately, the sintering process, while improving the mechanical properties of the cores, diminished the electrical performance of the inductors. This is mainly attributed to a phase transition from γ-Fe_2_O_3_ to α-Fe_2_O_3_ upon heating.^41,50,51^ XRD (Figure S7) indicated the presence of the crystalline γ-Fe_2_O_3_ phase for the pristine IOP powder and air-dried cores. However, the cores sintered at 600 and 800 °C only showed the presence of the α-Fe_2_O_3_ phase. Interestingly, the cores sintered at 400 °C exhibited a combination of the γ-Fe_2_O_3_ and α-Fe_2_O_3_ phases. These phase changes also support the observed color changes that occur when these samples are sintered (Figure 3). The α-Fe_2_O_3_ phase has a lower magnetic permeability and higher magnetic anisotropy than γ-Fe_2_O_3_, resulting in a lower inductance and higher magnetic losses in these inductors.^50^ Thus, there is a trade-off between mechanical and electrical properties for these IOP inductor cores. It is therefore crucial to select an optimum sintering temperature to achieve sufficient mechanical properties of the inductor core with as little detriment to the electrical properties as possible. For the inductors reported herein, 400 °C is the most preferred sintering temperature due to the good balance between electrical and mechanical properties. A more optimized sintering process may lead to further improvements and will be the subject of future investigations.

**Figure 7:**
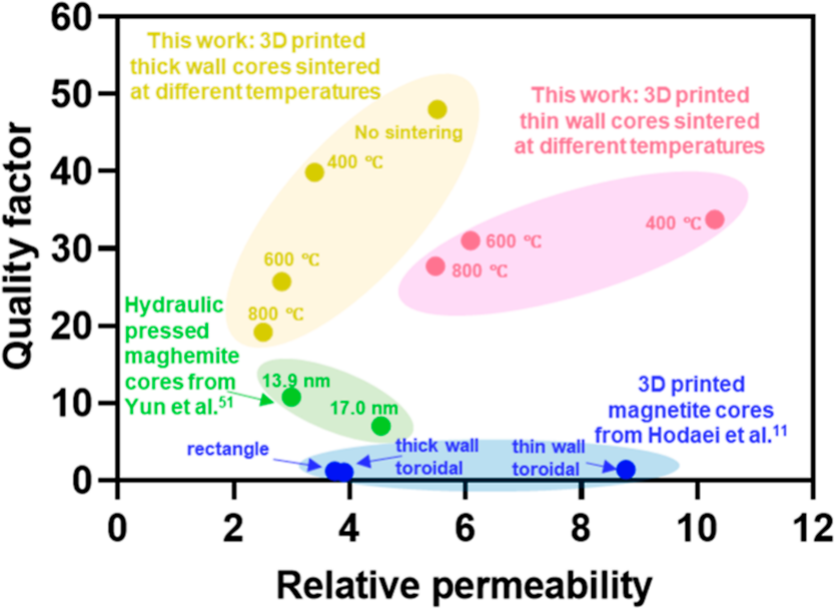
Ashby plot comparing the relative permeability of the 3D printed IOP core inductors reported herein to those reported by other groups. Quality factor and relative permeability values were calculated as described in the Supporting Information. Different categories of inductors are grouped as follows: yellow dots represent thick-walled toroidal cores from this work; red dots represent thin-walled toroidal cores from this work; and green dots represent toroidal cores prepared using a hydraulic press with different sized maghemite particles. Reproduced from ref (51). Copyright 2014 American Chemical Society; and blue dots represent 3D printed magnetite cores with different shapes. Reproduced from ref (11). Copyright 2018 American Chemical Society.

The performance of the inductors reported herein and other iron oxide-based inductors from published reports^11,51^ at 10 MHz were compared following the methodology described in the Supporting Information and Table S3. The 3D-printed IOP inductors from this work have higher *Q* factors (20–50) than other inductors whose *Q* factors are approximately 10 (hydraulic pressed maghemite inductors) and 1 (3D printed magnetite inductors) (Figure 7).^11,51^ For reference, a *Q* factor of 1 is too low for practical applications and means that nearly 50% of energy is lost per oscillation. Thus, magnetite inductors are not suitable for high-frequency (10 MHz) applications. The high energy loss in magnetite cores is mainly caused by hysteresis and eddy current loss.^43,46^ In contrast, the core materials described herein are maghemite (no sintering) and mixtures of maghemite and hematite (400 °C) and hematite (600 and 800 °C). Hematite has a higher hysteresis loss than maghemite, which results in the lower *Q* factors observed.^50,52^ The *Q* factor of the previously reported inductors with hydraulicly pressed maghemite cores is approximately 1/5 of the IOP thick-walled air-dried inductor and 1/4 of the 3D printed IOP inductors sintered at 400 °C. This is because the oxidation of iron oxide from magnetite to maghemite did not fully occur for the hydraulicly pressed maghemite cores and other residual phases such as magnetite, hematite, and wüstite were present.^51^ In addition, cracks and gaps were also observed for the pressed maghemite cores, which could also increase loss due to eddy currents and hysteresis.^47,49^

## Conclusions

PGMA_*x*_ (where *x* = 20 to 98) was successfully synthesized via RAFT polymerization and used as an additive for formulating IOP inks. The addition of PGMA_*x*_ significantly reduced the viscosity of high-concentration IOP suspensions, even when added in minute quantities (<1% w/w). Systematic investigations were conducted to determine the optimal conditions for IOP suspension rheology, with a formulation consisting of 0.25% (w/w) PGMA_98_ dosage, based on IOP concentration, at pH 10 being optimal. Under these conditions, inks comprising 70% w/w IOPs were 3D-printed to form thin- and thick-walled toroidal cores and rectangular blocks, which were subsequently sintered to enhance their mechanical properties. A high aspect ratio cylinder was printed successfully, demonstrating the excellent shape retainability of this IOP ink formulation, and additional structures were printed to demonstrate the ability of this IOP ink for rapid prototyping. In addition, the inks demonstrated good printability onto uneven substrates such as cardboard, paper, and nitrile rubber. One limitation of this study was that the 3D printer used restricted the maximum IOP concentration to ≤70% w/w, with rheological analysis indicating that higher concentration inks could be printable with the appropriate equipment. SEM analysis revealed that particle size growth and a more homogeneous microstructure were the primary contributors to the improvement of mechanical properties upon sintering. However, sintering also induced a phase transformation from γ-Fe_2_O_3_ to α-Fe_2_O_3_ that led to a decrease in electrical performance. Despite this trade-off, the fabricated inductors exhibited superior electrical properties, including the highest relative permeability and *Q* factor, compared to other iron oxide-based inductors reported in the literature.^11,51^ This is partly because γ-Fe_2_O_3_ has low coercivity, ultrahigh electrical resistivity, and good thermal stability when compared to other kinds of iron oxide. This comprehensive approach, spanning additive synthesis, ink preparation, device fabrication, and electrical characterization, demonstrates the promising potential of high ceramic loading 3D printable inks with customized, low-dosage additives. Furthermore, this innovative method paves the way for further advancements in the fabrication of complex, high-performance inductors and other functional magnetic devices through 3D printing technologies.

## Supporting Information Available

Additional experimental methods; reaction scheme for PGMA_x_ polymerization; ^1^H NMR and GPC characterization of PGMA_98_; summary of PGMA_x_ samples; TGA, zeta potential, and DLS analysis of PGMA_98_-containing IOP samples; summary of mechanical properties of 3D-printed samples; SEM particle size distributions; XRD data; photos of the I&J7300-LF 3D printer and 3D printed structures and letters on different substrates; photographs of mechanical testing instruments; and details and summary table comparing electrical performances (PDF)
