# Supplementary material for: Polymer-Assisted 3D Printing of Inductor Cores
Source: ACS Appl Mater Interfaces. 2024 Feb 13;16(8):10764–73. doi: 10.1021/acsami.3c18956 (PMC10910495; doi:10.1021/acsami.3c18956)
Supplement: Supplementary file 1 — am3c18956_si_001.pdf [file am3c18956_si_001.pdf]

‘**Supporting Information for:**

# Polymer-assisted 3D Printing of Inductor Cores

*Zhidong Luo,[a,b] Qi Yue,[a,b] Xueyuan Li,[a,b] Yuchen Zhu,[a,b] Xuzhao Liu[a,b] and Lee A. Fielding*[a,b]*

*a. Department of Materials, School of Natural Sciences, University of Manchester, Oxford Road, Manchester, M13 9PL, U.K.*

*b. Henry Royce Institute, The University of Manchester, Oxford Road, Manchester, M13 9PL, U.K.*

** Corresponding author: lee.fielding@manchester.ac.uk*

## Additional Experimental Details

**$^1$H nuclear magnetic resonance (NMR).** $^1$H NMR spectra were recorded on a Bruker Avance III 400 MHz spectrometer with 128 scans averaged per spectrum at 25 °C.

**Gel permeation chromatography (GPC).** Molar mass distributions were measured using an Agilent 1260 Infinity II GPC system equipped with two Polymer Laboratories PLgel mixed D columns. HPLC-grade DMF containing 10 mM LiBr at 60 °C was used as an eluent at a flow rate of 1.0 mL $min^{-1}$. A refractive index detector was used to assess molar mass distributions using poly(methyl methacrylate) (PMMA) individual narrow standards calibration standards.

**Zeta potential and dynamic light scattering (DLS) measurements.** A Malvern Zetasizer Ultra instrument equipped with a He-Ne solid-state 633 nm laser and a scattered light detector at a constant angle of 173° was used for both zeta potential and DLS measurements. The iron oxide nanoparticle (IOP) concentration used was 0.1% w/w, and the samples were diluted using 1 mM KCl aqueous solution. The pH of samples was adjusted with 0.1 M KOH, 0.01M KOH, 0.1M HCI and 0.01M HCI. All measurements were conducted at 25 °C, and all data were averaged over three measurements.

**Thermogravimetric analysis (TGA).** TGA was carried out using an SDT 650 simultaneous thermal analyzer (TA Instruments). To prepare samples, 0.2 g IOPs were added into 20 g deionized water containing different concentrations of PGMA, followed by 5 min mechanical stirring and 30 min ultrasonication. Then, mixtures were centrifuged at 6000 rpm for 30 min. The sediments were collected and redispersed in 30 mL deionized water followed centrifugation at 6000 rpm for 30 min. Afterwards, the sediment was collected and dried at 60 °C for 48 h. The obtained dried powders were used for TGA. TGA was conducted in an air atmosphere by heating from 25 °C to 800 °C at 10 °C $min^{-1}$. The mass of adsorbed additives was calculated by comparing the weight loss between polymer coated IOPs and pristine IOPs.

**X-ray diffraction (XRD).** XRD measurements were conducted using a Bruker D8 DISCOVER instrument with a Cu target ($\lambda$ = 1.5418 Å) at a generating voltage of 40 kV, and current of 40 mA. The scattering angle was varied from 20 to 80° in the $\theta$−2$\theta$ scan mode with a 0.01° step size and 2 s dwell time. During testing, samples were rotated at a speed of 10 rpm.

**Supporting Figures and Tables**

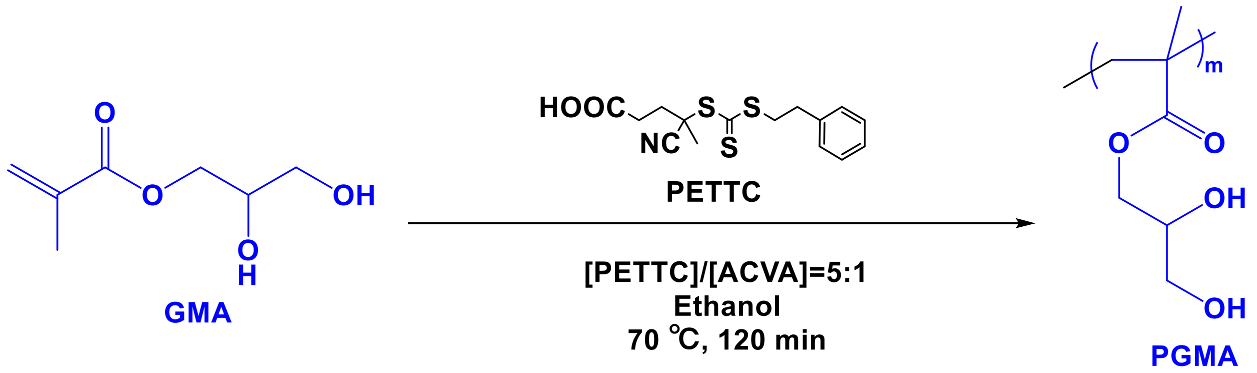


**Figure S1.** Synthesis of $PGMA_m$ *via* RAFT solution polymerization in ethanol at 70 °C.

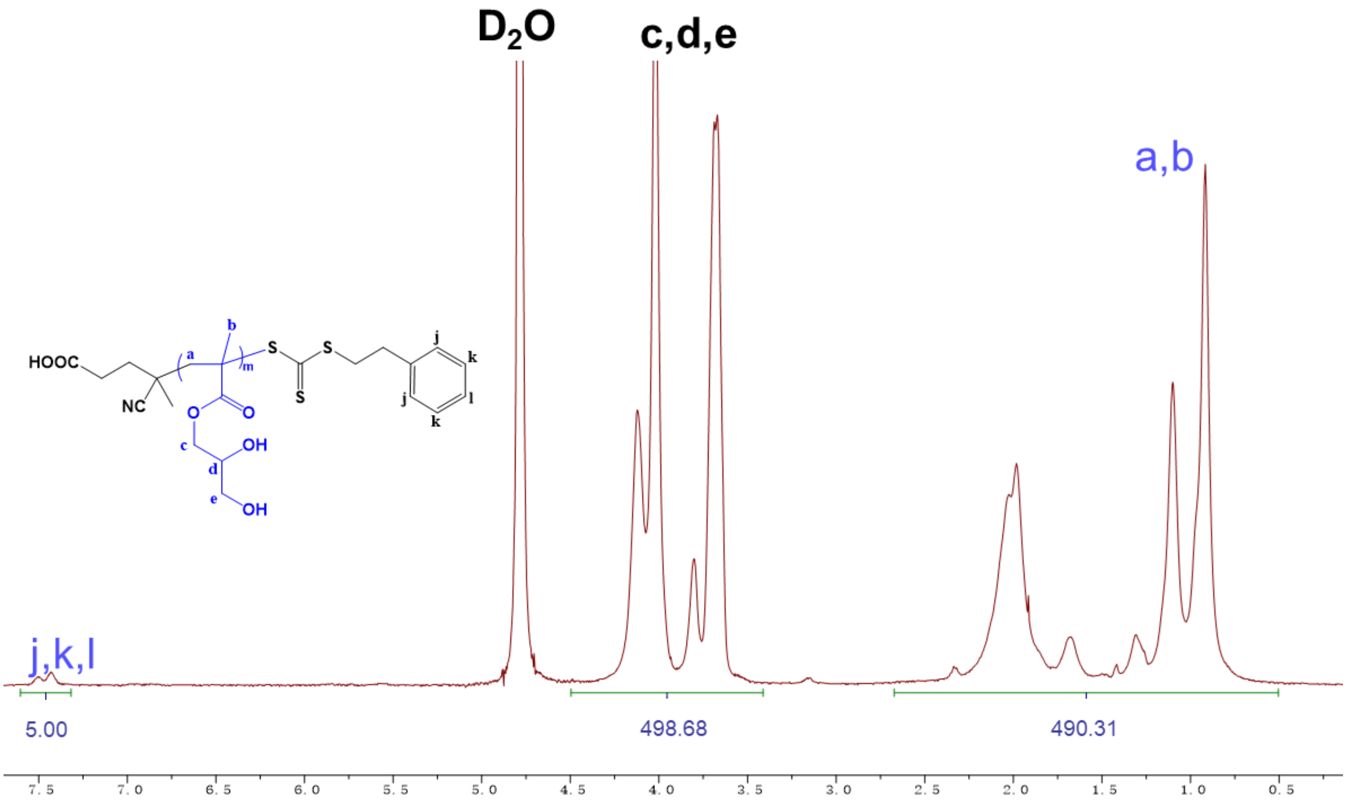


**Figure S2.** Assigned $^1H$ NMR spectrum of $PGMA_{98}$ in $D_2O$.

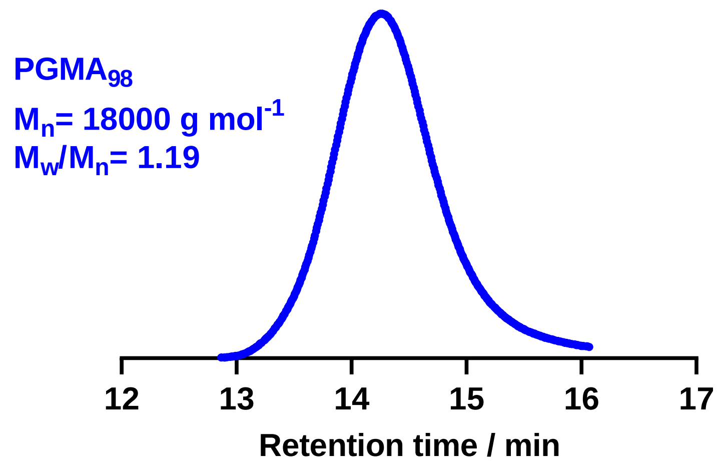


**Figure S3.** GPC chromatogram for $PGMA_{98}$. DMF was used as the eluent. $M_n$ and $M_w/M_n$ values were determined using poly(methyl methacrylate) calibration standards.

**Table S1.** Summary of $PGMA_x$ samples

| **Entry** | **Composition**$^a$ | $M_n$ **/ g mol**$^{-1}$ | $M_w/M_n$ $^c$ |
|---|---|---|---|
| **1** | $PGMA_{20}$ | 3540$^b$ | |
| **2** | $PGMA_{54}$ | 11500$^c$ | 1.21 |
| **3** | $PGMA_{65}$ | 12800$^c$ | 1.25 |
| **4** | $PGMA_{80}$ | 13100$^b$ | |
| **5** | $PGMA_{98}$ | 18000$^c$ | 1.19 |

*a*. Determined by $^1$H NMR end-group analysis.

*b*. Calculated using $M_n$ = repeat unit molar mass × degree of polymerization determined by NMR.

*c*. Measured by DMF GPC.

(a)

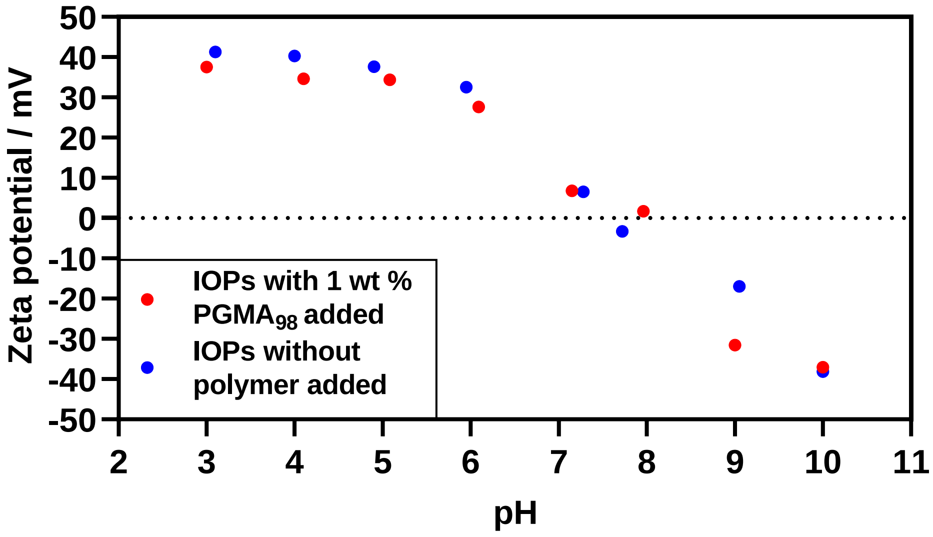


(b)

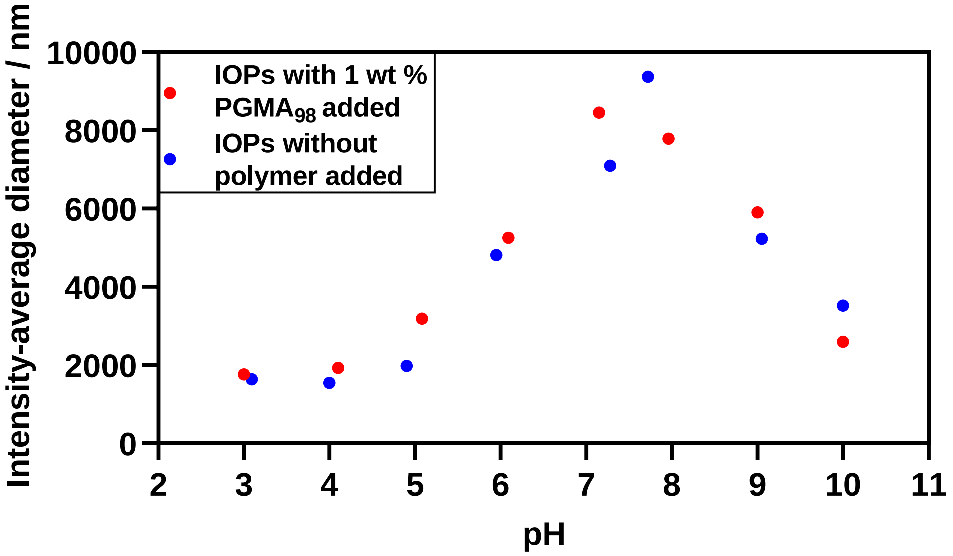


**Figure S4.** (a) Zeta potential and (b) intensity-average diameter as a function of pH obtained for iron oxide particles with 1% w/w $PGMA_{98}$, based on IOP concentration (red dots), and IOPs without additional polymer (blue dots). Measurements were conducted at an IOP concentration of 0.1% w/w in the presence of 1 mM KCl as a background electrolyte.

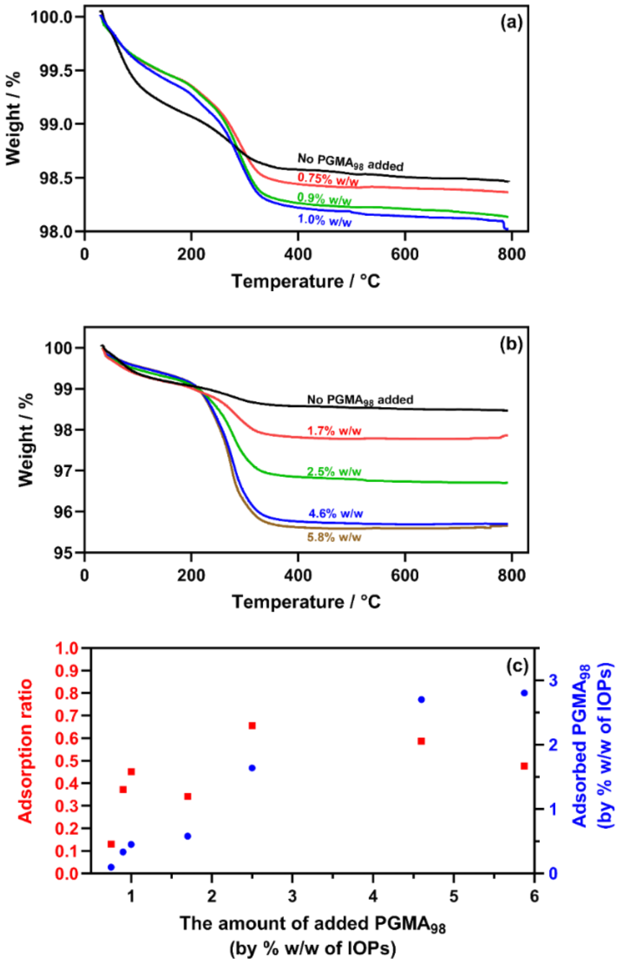


**Figure S5.** TGA data for IOPs with different amounts of $PGMA_{98}$ added IOPs. (a) Red, green, blue and black curves represent 0.75%, 0.9%, 1.0% and 0.0% w/w $PGMA_{98}$ added, respectively. (b) Red, green, blue, brown and black curves represent 1.7%, 2.5%, 4.6%, 5.8% and 0.0% w/w $PGMA_{98}$ added, respectively. (c) Amount of absorbed $PGMA_{98}$ (blue) and adsorption ratio (red) calculated from TGA curves. The adsorbed amount of $PGMA_{98}$ is the amount of the polymer adsorbed to IOPs based on the mass of IOPs. The amount of added $PGMA_{98}$ is the amount of $PGMA_{98}$ added based on the mass of IOPs present. The adsorption ratio is the mass of added $PGMA_{98}$ divided by the mass of absorbed $PGMA_{98}$, as detailed below.

**Calculation of the adsorption ratio and amount of absorbed $PGMA_{98}$**

$x$ is defined as the amount of added $PGMA_{98}$ based on the mass of IOPs present.
$M_{reference}$ is defined as the residual mass fraction after performing TGA on pristine IOPs, and was set as the reference.
$M_x$ is defined as the residual mass fraction after TGA of IOPs with varying amounts of $PGMA_{98}$ added.
The adsorbed amount of $PGMA_{98}$ was calculated using:

$$Adsorbed\ PGMA_{98} = \left(M_{reference} - M_x\right) \div M_{reference} \times 100 \quad (1)$$

and

$$\text{Adsorption ratio} = \text{Adorbed PGMA}_{98} \div x \quad (2)$$

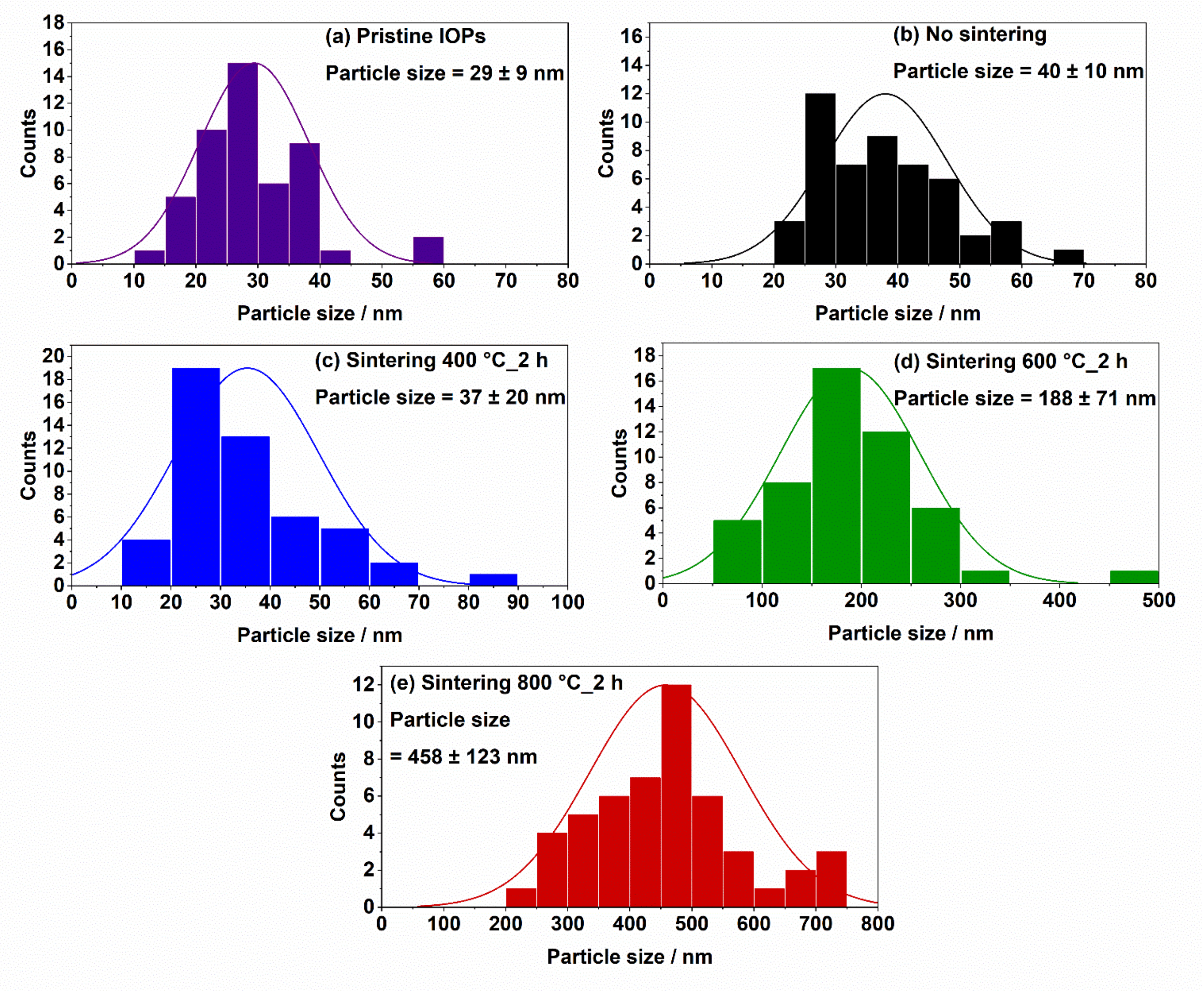


**Figure S6.** Particle size distribution histograms determined from SEM image analysis of (a) pristine IOP powder, and the outer surfaces of 3D printed thin-walled toroidal cores after being sintered at different temperatures: (b) no sintering, (c) sintering at 400 °C for 2 hours, (d) sintering at 600 °C for 2 hours and (e) sintering at 800 °C for 2 hours.

**Table S2.** Mechanical properties of 3D-printed IOP samples[a]

| Sintering Temperature[b] | Flexural Mechanical Properties[c] | | | Compressive Mechanical Properties[g] | | |
|---|---|---|---|---|---|---|
| | Flexural Stress[d] / kPa | Flexural Strain[e] / % | Flexural Modulus[f] / MPa | Compressive Stress[h] / kPa | Compressive Strain[i] / % | Compressive Modulus[j] / MPa |
| **Air Dried** | | | | 3490 | 8.6 | 3.5 |
| **400 °C** | 1750 | 0.34 | 458 | 11680 | 12.0 | 76 |
| **600 °C** | 3890 | 0.59 | 609 | 13880 | 7.0 | 157 |
| **800 °C** | 9830 | 0.08 | 11783 | 118210 | 15.4 | 832 |

*a*. Shapes for testing were printed using 70% w/w IOP at pH 10 with 0.25% w/w $PGMA_{98}$, based on IOP concentration. *b*. Sintering profiles are shown in Figure 4a. *c*. Measured using 3D-printed rectangular blocks. *d*. Stress at failure point in four-point flexural testing. *e*. Strain at failure point in four-point flexural testing. *f*. Obtained by fitting the slope between flexural stress and strain. *h*. Stress at failure point in compressive testing. *i*. Strain at failure point in compressive testing. *j.* Obtained by fitting the slope between compressive stress and strain.

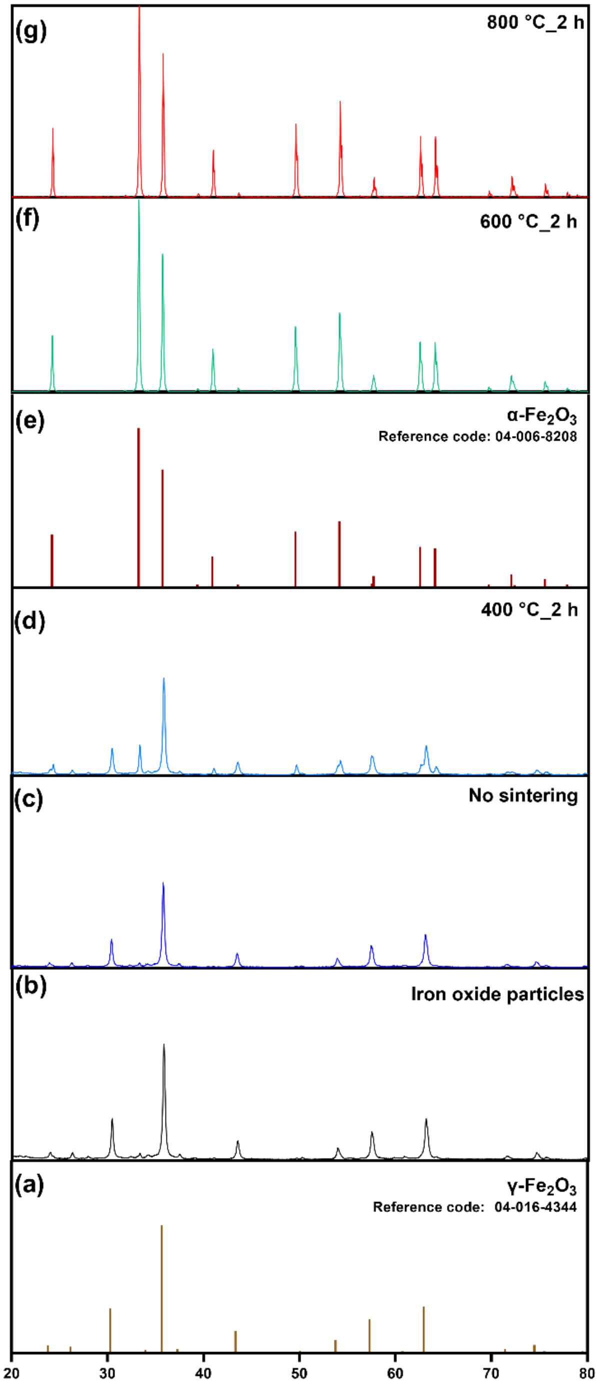


**Figure S7.** XRD patterns at room temperature and XRD reference cards. (a) Reference card of γ-$Fe_2O_3$, (b) pristine IOPs, (c) the printed sample without sintering treatment, (e) reference card of α-$Fe_2O_3$, and the printed samples sintered at (d) 400 °C, (f) 600 °C and (g) 800 °C for 2 hours. The ink used for printing contained 70% w/w IOPs and 0.25% w/w $PGMA_{98}$, based on IOP concentration.

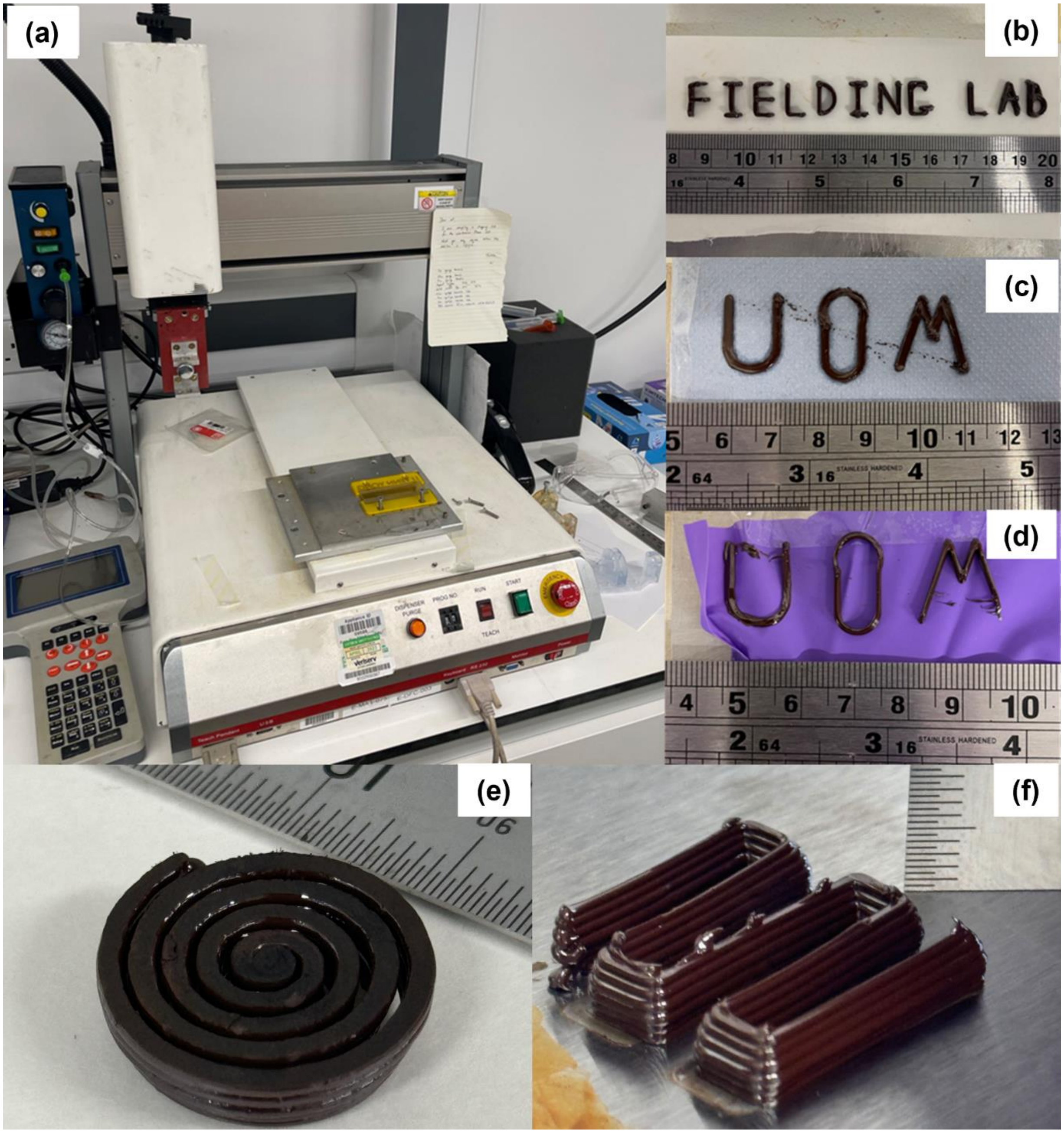


**Figure S8**. (a) Printing set-up of the I&J7300-LF 3D printer. The printer had a movable metal base, a movable head and pressure unit. 3D printed letters on (b) cardboard, (c) a piece of blue tissue paper, and (d) a piece of nitrile rubber glove to test the ink printability on different substrates at room temperature. (e) A 3D printed spiral-shaped structure (height × outer diameter × wall thickness = 6.4×20×1 mm) and (f) a 3D printed comb-like thin-walled structure (length × width × wall thickness = 20×20×1 mm). The printing speed was 8 mm $s^{-1}$.

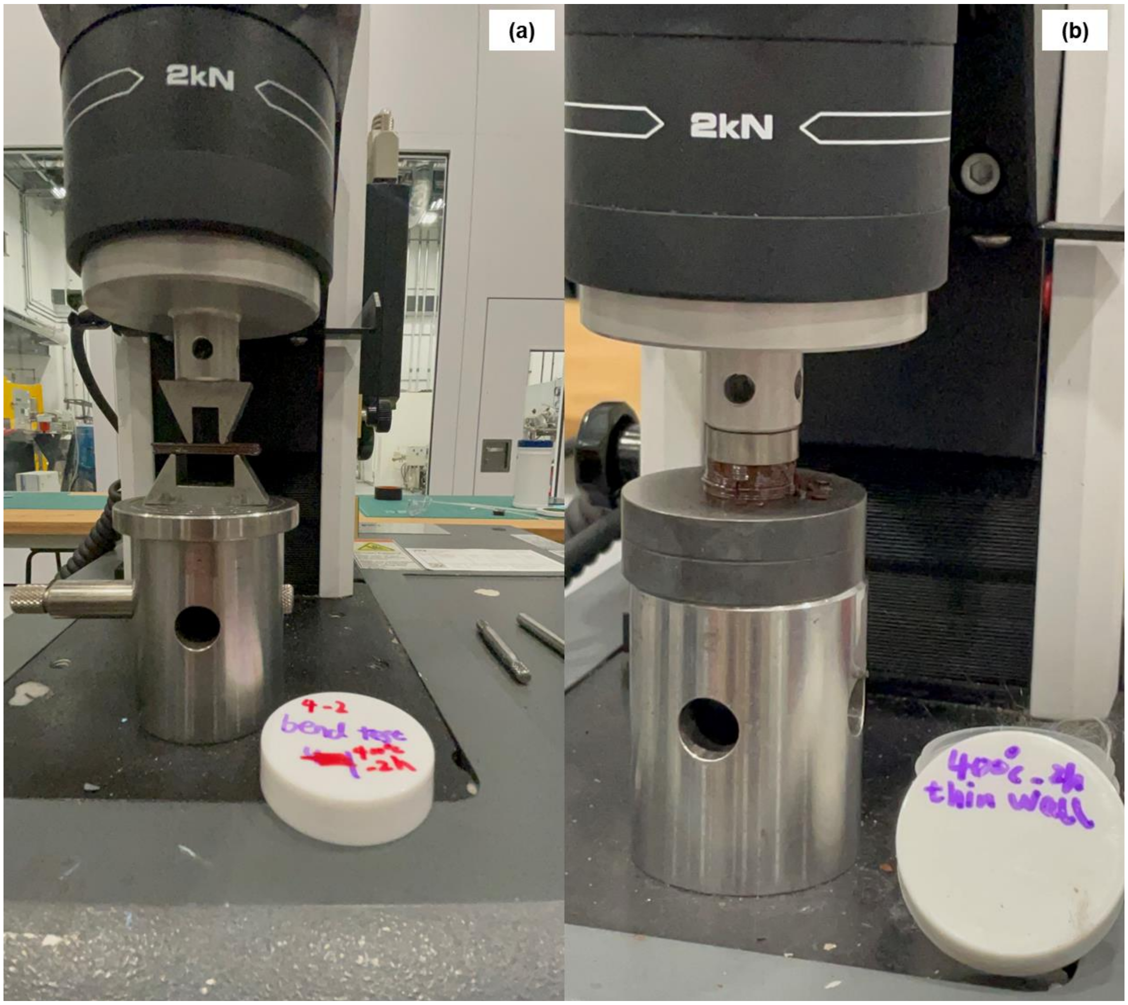


**Figure S9.** Mechanical test instrumentation. (a) Four-point flexural testing of a rectangular block (the sample was sintered at 400 °C for 2 hours); (b) compressive testing of a thin-walled toroidal core (the sample was sintered at 400 °C for 2 hours). All mechanical testing was conducted at room temperature.

**Determination of the relative permeability and Q factor**

The inductance of an inductor is defined by the following equation:

$$L = \frac{N^2 \mu_0 \mu_r A}{l} \tag{3}$$

where *L* is the inductance in Henry, *N* is the number of turns in the coil, $\mu_0$ is the permeability of free space ($1.26\times10^{-6}$ H $m^{-1}$), *A* is the area of each turn in the coil in square meters, *l* is the length of the coil in meters, and $\mu_r$ is the relative permeability of the inductor. The relative permeability is a fundamental property of magnetic cores that is independent of the geometry and dimensions of the core. This makes it a more reliable indicator of core performance than inductance, which depends on the physical dimensions and configuration of the core. Equation 3 is therefore converted to equation 4 for relative permeability calculations.

$$\mu_r = \frac{lL}{N^2 \mu_0 A} \tag{4}$$

The inductance and geometry of different inductors reported in this work and in the cited literature is shown in Table S3. In this work and that of Hodaei et al.[1] the coil was wound around the inductor cores, so the cross-sectional area of the core was taken as the area of each turn to calculate the relative permeability. In the report by Yun et al., [2] toroidal cores were first integrated within a laser-cut plastic bobbin that were then wound in wire coils. The cross-section of the bobbin was larger than the cross-section of the cores, so the cross-section of the core could not be determined from the area of each turn. Another method was therefore used to calculate the relative permeability, as shown in equation 5:

$$\mu_r = \frac{L}{L(air\ core)} \tag{5}$$

Where *L* is the measured inductance of the maghemite core inductor and *L(air core)* is the calculated inductance value for an inductor with an air core with the same geometry. The value of *L(maghemite core)* and *L(air core)* is shown in Table S3.

**Table S3.** Electrical properties and geometries of different iron oxide-based inductors.

| Sample | *L* / nH | *A* / cm$^2$ | *l* / cm | *N* | *L(air core)* / nH | $\mu_r$ | Quality factor |
|---|---|---|---|---|---|---|---|
| Thick wall inductor_ no sintering[a] | 1300 | 0.24 | 5.3 | 20 | | 5.5 | 47 |
| Thick wall inductor_400 °C[a] | 770 | 0.24 | 5.3 | 20 | | 3.4 | 40 |
| Thick wall inductor_600 °C[a] | 640 | 0.24 | 5.3 | 20 | | 2.8 | 26 |
| Thick wall inductor_800 °C[a] | 570 | 0.24 | 5.3 | 20 | | 2.5 | 19 |
| Thin wall inductor_400 °C[a] | 690 | 0.08 | 6.0 | 20 | | 10.3 | 34 |
| Thin wall inductor_600 °C[a] | 410 | 0.08 | 6.0 | 20 | | 6.1 | 31 |
| Thin wall inductor_800 °C[a] | 370 | 0.08 | 6.0 | 20 | | 5.5 | 28 |
| Thin wall magnetite inductor[b] | 8000 | 0.12 | 6.0 | 60 | | 8.8 | 1.4 |
| Thick wall magnetite inductor[b] | 15000 | 0.32 | 3.8 | 60 | | 3.9 | 1 |
| Rectangle inductor[b] | 11000 | 0.15 | 2.4 | 60 | | 3.7 | 1.4 |
| Maghemite inductor_17.0 nm[c] | 500 | | | | 110 | 4.6 | 7.1 |
| Maghemite inductor_13.9 nm[c] | 300 | | | | 100 | 3.0 | 11 |

*a*. This work. *b*. Work of Hodaei et al.[1] *c*. Work of Yun et al.[2]

**References**


(1) Hodaei, A.; Akhlaghi, O.; Khani, N.; Aytas, T.; Sezer, D.; Tatli, B.; Menceloglu, Y. Z.; Koc, B.; Akbulut, O. Single Additive Enables 3D Printing of Highly Loaded Iron Oxide Suspensions. *ACS Appl Mater Interfaces* **2018**, *10* (11), 9873–9881.

(2) Yun, H.; Liu, X.; Paik, T.; Palanisamy, D.; Kim, J.; Vogel, W. D.; Viescas, A. J.; Chen, J.; Papaefthymiou, G. C.; Kikkawa, J. M.; Allen, M. G.; Murray, C. B. Size- and Composition-Dependent Radio Frequency Magnetic Permeability of Iron Oxide Nanocrystals. *ACS Nano* **2014**, *8* (12), 12323–12337.
